# Exploring Substance Use and HIV Treatment Factors Associated with Neurocognitive Impairment among People Living with HIV/AIDS

**DOI:** 10.3389/fpubh.2014.00105

**Published:** 2014-08-11

**Authors:** Jennifer M. Attonito, Jessy G. Dévieux, Brenda D. G. Lerner, Michelle M. Hospital, Rhonda Rosenberg

**Affiliations:** ^1^Department of Health Promotion and Disease Prevention, Robert Stempel College of Public Health & Social Work, Florida International University, Miami, FL, USA; ^2^Department of Psychology, College of Arts and Sciences, Florida International University, Miami, FL, USA; ^3^Community-Based Intervention Research Group, School of Integrated Science and Humanity, Florida International University, Miami, FL, USA

**Keywords:** HIV, neurocognitive, alcohol, marijuana, Color Trails Test

## Abstract

Neurocognitive (NC) impairment remains prevalent among people living with HIV (PLWH) and may be exacerbated by alcohol and drug use. This cross-sectional study assesses the degree to which alcohol and other drug use, time from HIV diagnosis to treatment, and years living with HIV affect three areas of NC functioning among HIV-seropositive adults. NC functioning in 370 PLWH living in Miami, FL was assessed using the Auditory Verbal Learning Test, the Short Category Test, Booklet Format, and the Color Trails Test 2 (CTT2). Participants reported the number of days using alcohol, marijuana, and cocaine over the previous 3 months, the number of known years living with HIV and length of time from HIV diagnosis to seeking care. Bivariate linear regression and multivariate linear regression were used to test associations between independent and dependent variables. Mean scores on NC measures were significantly lower than published norms; 39% of participants scored ≥1 standard deviation below normative sample means on >2 NC tests. No significant associations were found between alcohol or cocaine use and any NC measure. Years living with HIV was associated with CTT2 in the bivariate analysis (β = 1.031; *p* = 0.007). In multivariate analysis, each day of marijuana use and years living with HIV were associated with a 0.32 (*p* = 0.05) point and 1.18 (*p* = 0.03) points poorer performance score on the CTT2, respectively. Results suggest that both marijuana use and duration of HIV infection may affect cognitive functioning among PLWH in ways that may impair their ability to follow important treatment guidance.

## Introduction

Since the introduction of highly active antiretroviral treatment (ART), the incidence of HIV-associated dementia has declined from 10 to 15% to rates of 2–5% in people living with HIV (PLWH) (1–3). However, milder forms of HIV-associated neurocognitive disorders (HAND) continue to be reported in up to 40% of this population, in part because HIV-positive individuals are living longer with the disease (4–8).

HAND, ranging from mild impairment to profoundly disabling HIV-associated dementia (7), is mostly seen in advanced stages of HIV disease but can occur even in PLWH who have medically asymptomatic HIV infection (9–11). While increased time living with the virus, particularly during the acute phase of HIV infection, is associated with neurocognitive (NC) impairment, HAND has been observed even among PLWH who have consistently maintained low-to-undetectable viral loads on ART (12, 13). Early initiation of ART is known to reduce the risk of developing HAND (14, 15). At the time of this study, treatment guidelines recommended that all HIV-infected adults with a CD4 count of <350 be prescribed ART.

HAND can express clinically as impairment in episodic memory, information processing, attention, and executive functions (16). In patients presenting with even milder forms of HAND, quality of life can be greatly affected, with individuals suffering from difficulties in ability to perform activities of daily living, personal health care management, medication management, and risk behavior reduction (6, 17, 18). Mild or asymptomatic NC impairment is known to be more prevalent than the more debilitating HAND diagnoses (19, 20); however, even when NC impairment is asymptomatic, HAND may still be associated with functional problems such as poorer employment capacity and lower symptom reporting related to lower self-awareness (1, 19, 21).

Abuse of alcohol and other drugs frequently co-occurs with HIV and may further impair NC functioning (22, 23). HIV infection and heavy drug or alcohol use have been observed to have synergistically negative effects on NC functioning and the progression of HAND (24–26). Impaired verbal and auditory working memory, and enhanced cognitive impulsivity have been observed (27, 28). These cognitive functions are associated with the higher-order thinking required to conduct safer sex practices and health management behaviors such as ART adherence (17, 24, 29, 30).

It is important to understand specific domains of impairment associated with exposure to HIV and the use of commonly abused substances such as alcohol, marijuana, and cocaine in order to deliver targeted and effective clinical care, as well as HIV risk reduction and treatment adherence interventions. Using structural equation modeling (SEM), this study sought to identify direct relationships between exposure to HIV and degree of alcohol and drug use upon several major domains of NC functioning including memory, information processing, attention, and executive function. It was expected that longer time living with HIV, longer time from HIV diagnosis to seeking care, and greater use of substances would reduce performance on several NC measures. Outcomes may help identify areas of cognitive functioning affected by substance abuse and HIV in order to improve ways in which health care information is delivered by clinicians and retained by patients.

## Materials and Methods

### Study design

This study employed a cross-sectional design, utilizing baseline data gathered between 2009 and 2012 as part of a prospective randomized controlled trial for HIV-positive adult alcohol users. Participants (*N* = 370) were recruited from 13 community-based organizations (CBOs) in densely populated, multicultural, low-income, urban areas of South Florida, primarily Miami-Dade County, with high rates of substance abuse, HIV, violence, and poverty. The CBOs were among the largest in the local area providing substance abuse treatment (inpatient or outpatient) and mental health services to HIV-positive men and women. Recruitment settings were selected from a wide range of non-academic institutions, specialty and primary care, public and private facilities; however, 23.5% of participants were in residential substance abuse treatment at the time of data collection. The inclusion criteria were: being 18 to 60-years-old; being HIV-positive; having consumed any alcohol in the past 3 months; having a history of alcohol abuse or dependence within the past 2 years; facility in English; and currently not showing overt signs of any major psychiatric disorder. Participants in the parent study were randomized to a group level, 8-week intervention to reduce substance abuse, and sex risk among PLWH or to a similarly administered health comparison group providing standard of care content. The research protocol was approved by the Institutional Review Board of Florida International University and all participants provided signed informed consent prior to participating in the study.

Assessment methods consisted of: (1) computer-assisted personal interview (CAPI); (2) audio computer-assisted self-interview for subjective sensitive topics (ACASI); (3) paper and pen as specified for neurological measures; and (4) Timeline Follow-Back (TLFB).

### Measures

“Years living with HIV” was measured by asking the year, they received their first HIV-positive test. This value was subtracted from the year of intake to calculate number of years living with HIV.

Time from HIV diagnosis to seeking medical care was an ordinal item from the Community Programs for Clinical Research on AIDS [CPCRA; (31)] asking “How soon after your positive test for HIV did you first go for medical care for your HIV?” Response options were: (1) within 6 weeks; (2) 6–12 weeks; (3) 3–6 months; (4) 6–12 months; (5) more than a year; and (6) I have not gone for medical care for my HIV. This final response option did not provide an interval time measurement. Two participants selecting option (6) had been diagnosed at least 1 year before interview and were classified as option (5) and one participant had been diagnosed recently so this response was coded as “missing.” It should be noted that this question did not ask when treatment was initiated, only when it was sought.

Alcohol, marijuana, and cocaine use were assessed by TLFB to provide a continuous 3 months history for intensity of drug and alcohol consumption. These variables were measured: total number of “heavy drinking days” (defined as ≥5 drinks), total number of marijuana use days, and total number of cocaine use days. Up to 3 months recall of alcohol and other drug use has proven to yield reliable data (32). TLFB has strong agreement with other measures of substance use and has reliability measures ranging from 0.75 to 0.90 (33).

Three neuropsychological tests were administered to derive scores in various cognitive domains, selected for importance to behaviors associated with maintaining health and reducing risk of transmission among PLWH. For example, information processing and memory are related to comprehending, retaining, and applying instructions such as medical advice and medication dosing (34). The tests selected have been used in previous studies with HIV-positive users of alcohol, marijuana, and cocaine and have well-developed norms, high reliability, and high validity (24, 35–39).

#### Auditory Verbal Learning Test, University of California Los Angeles/World Health Organization Version

Fifteen words were read to the participant, requesting both immediate and longer-term recall (34, 38). Scores for immediate recall, delayed recall, and percent of words retained can be derived. This study utilized the total of immediate recall scores for trials 1–5, with higher scores indicating greater functioning. This instrument demonstrated high test-retest reliability, with alpha scores ranging from 0.51 to 0.72 (34).

#### The Color Trails Test 2, Form A (CTT2)

CTT-2 shows two sets of 1–25 numbered circles, and using a pen, the participant must quickly connect the numbers in order, alternating between pink and yellow (34, 40, 41). This analysis used the raw time in seconds the participant required to complete the test, with higher scores indicating poorer functioning. The test is sensitive to a variety of neurological impairments and processes (42) and, because it is entirely numeric, it is considered culturally unbiased (36). Additionally, the instrument has shown strong agreement with other cognitive assessments among PLWH (36, 43) and has displayed good temporal stability with test-retest reliability between 0.85 and 1.00 (41).

#### The Short Category Test booklet format

This assesses problem-solving ability, requiring the examinee to determine the organizing principle behind a series of visually presented stimuli, based on external feedback from the test administrator (44, 45). This study uses the total raw error score derived, with higher scores indicating poorer functioning. Test-retest coefficients have varied from 0.60 to 0.96 depending upon the severity of impairment in the sample (44).

### Analytic strategy

A hypothesized directional model was constructed to assess the degree to which independent relationships exist between HIV exposure, alcohol use, marijuana use, and cocaine use on the outcome of NC functioning as indicated by three different cognitive performance measures.

Means, standard deviations, skewness, and kurtosis levels were calculated for all continuous variables; frequencies were generated for dichotomous and categorical variables. In order to gain an understanding of the overall degree of NC impairment in this sample, one-sample *t*-tests were used to compare mean raw NC scores with normative scores on each instrument. Raw scores were square root transformed, with confidence intervals representing the difference between the raw, transformed scores, and square roots of the normative scores. Norms for NC measures were obtained from professional manuals provided for the instruments (41, 46, 47). In addition, using chi-square analysis, participants were dichotomized – those who scored >1 standard deviation below the means of normative samples on at least two NC assessments versus those with higher NC performance – and cross tabulated on whether they reported taking prescribed ART. Raw NC scores were used in bivariate and multivariate regression analyses; age, gender, and educational level were entered as covariates to control for their possible impact upon NC scores. Bivariate regression analyses assessed relationships between each independent and dependent variable using SPSS version 21. Maximum Likelihood framework was invoked in MPlus version 5.1 (48) to accommodate non-normality in the multivariate, SEM analysis (49). Approximately 8% of data was missing randomly across variables. With no systematic patterns observed in the missing data, a full information maximum likelihood method was used to accommodate the missing data in the SEM analysis. To assess non-model-based outliers, leverage indices were examined for each participant based on their multivariate profile for the variables included in the model analyses. No observations had a leverage score four times greater than the mean leverage (0.09) in the sample data. After addressing all diagnostic statistics, the final hypothesized model was analyzed using Mplus; strengths of association were reported.

## Results

The mean age of participants was 44.79 years; most (63.5%) were men and identified as black (76.2%). Most (55.7%) of participants reported at least high school completion; few (8.2%) were employed. The mean number of years living with HIV was 12.2. While most (58.4%) sought medical care within 6 weeks of receiving a positive HIV test, 24.6% of participants waited over a year before seeking care (Table 1). Although not included in the tested model, the majority (76.7%) reported that they were currently taking ART; 80% reported perfect (100%) treatment adherence; and 45% had an undetectable viral load when last tested. Use of marijuana in this cohort was substantially lower than use of alcohol and cocaine; however, more than half of all participants reported some marijuana use.

**Table 1:** **Sociodemographic characteristics of participants at baseline (*N* = 370)**.

	Mean (SD) *N* (%)	Skewness/Kurtosis
Age	44.79 (7.3)	−0.56/−0.13
Gender (male)	233 (63.5)	−0.56/−0.69
Hispanic ethnicity (yes)	53 (15)	1.97/1.90
Race		
Black	266 (76.2)	−1.24/−0.47
White	50 (14.3)	2.05/2.20
Years education		0.41/−0.17
<HS diploma	157 (44.3)	–
≥HS diploma	198 (55.7)	–
Employed (Yes)	29 (8.2)	3.07/7.45
Years since HIV diagnosis	12.15 (7.5)	0.20/−1.01
Time from HIV diagnosis to treatment		0.65/−1.41
Within 6 weeks	206 (58.4)	–
6–12 weeks	13 (3.7)	–
3–6 months	22 (6.2)	–
6 months–1 year	25 (7.1)	–
1 year+	87 (24.6)	–
Taking ART (yes)	273 (76.7)	−1.27/−0.40
Currently in residential substance treatment	84 (23.5)	0.66/−1.27
Days using in last 3 months
Alcohol	15.77 (23.33)	1.86/2.83
Marijuana	9.29 (20.68)	2.84/7.59
Cocaine	15.68 (23.89)	1.89/2.80
Neurocognitive functioning		
CTT2	116.31 (49.82)^a^	1.71/4.97
SCT	38.09 (15.58)^a^	−0.38/−0.86
AVLT	43.44 (9.63)^a^	−0.03/−0.22

*^a^Raw scores reported. All sample means are significantly different from normative sample at *p* < 0.01*.

*Normative scores used for comparison testing represented mean age and education for this sample. Skewness and Kurtosis values for neurocognitive instruments reflect distribution of scores prior to square root transformation*.

Single sample *t*-tests comparing the mean raw NC scores with normative scores on each instrument showed that performance for the study group was significantly poorer (all *p* < 0.001) than observed in healthy samples as follows: auditory verbal learning test (AVLT), *t* = −16.25 [95% CI = −0.67, −0.52]; CTT2, *t* = 6.65 [95% CI = 0.53, 0.97]; and short category test booklet format (SCT), *t* = 11.52 [95% CI = 0.66, 0.93]. Over a third (39%) of the study population scored at least one standard deviation below the mean for normative samples on at least two NC tests, consistent with clinically significant NC impairment (7). No association was observed between the higher and lower performing groups based upon whether they reported being on prescribed ART (χ^2^ = 0.021, *p* = 0.88).

**Figure 1:**
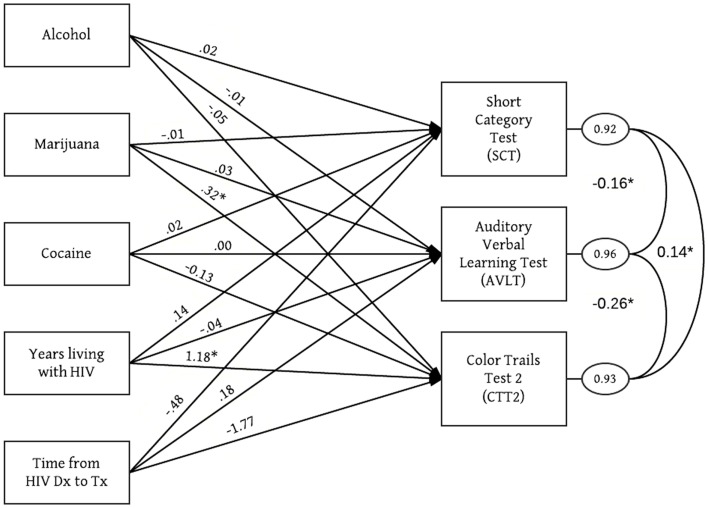
**Significant regression paths among measured variables in the structural equation model among HIV-positive alcohol abusers (*N* = 370)**. Model included age, gender and education level as covariates. Regression coefficients (represented as one-way arrows) are unstandardized. *p < 0.05.

Bivariate analyses (Table 2) identified one significant relationship: years living with HIV was associated with poorer scores on the CTT2 (β = 1.031; 95% CI: 0.285, 1.777; *p* = 0.007). After examining normality, outliers and patterns of missing data, the final multivariate model was tested. Figure 1 and Table 3 present significant and non-significant unstandardized path coefficients of the final model. This was a just-identified model (χ^2^ = 0, 0 *df*; CFI = 1; RMSEA = 0; SRMR = 0). All exogenous variables were assumed to be correlated. Unstandardized path coefficients, standard errors and 95% confidence intervals are listed in Table 3. Residual error terms across three measures of NC functioning were correlated. Focused fit indices did not show evidence of Heywood cases. Standardized residual values were <2. The final model explained 8% of variance in memory, 7% of variance in information processing, and 4% of variance in executive functions. In the final model, two associations approached or achieved statistical significance. On average, for each day of marijuana use, the CTT2 score increased by 0.32 (*p* = 0.051), indicating poorer performance on this NC measure. Similarly, on average, each year living with HIV increased the CTT2 score by 1.18 (*p* = 0.03).

**Table 2:** **Bivariate regression statistics, 95% confidence intervals, and *p*-values (*N* = 370)**.

		β	95% CI	*P*
Alcohol days^a^	AVLT^f^	− 0.005	−0.049, 0.039	0.82
	CTT2^g^	− 0.137	−0.386, 0.111	0.28
	SCT^h^	− 0.017	−0.089, 0.056	0.65
Marijuana days^b^	AVLT	0.009	−0.044, 0.063	0.73
	CTT2	0.275	−0.024, 0.573	0.07
	SCT	− 0.049	−0.137, 0.038	0.27
Cocaine days^c^	AVLT	0.011	−0.032, 0.053	0.63
	CTT2	− 0.172	−0.413, 0.069	0.16
	SCT	0.027	−0.033, 0.097	0.46
Years of HIV infection^d^	AVLT	− 0.075	−0.208, 0.059	0.27
	CTT2	1.031	0.285, 1.777	0.007
	SCT	0.096	−0.124, 0.316	0.39
Time from HIV diagnosis to seeking medical care^e^	AVLT	− 0.045	−0.570, 0.480	0.87
	CTT2	0.384	−2.509, 3.277	0.79
	SCT	− 0.333	−1.192, 0.526	0.447

*^a^Number of days using alcohol in past 3 months*.

*^b^Number of days using marijuana in past 3 months*.

*^c^Number of days using cocaine in past 3 months*.

*^d^Duration of known HIV infection in years*.

*^e^Time interval from diagnosis of HIV infection to seeking medical care*.

*^f^Auditory Verbal Learning Test*.

*^g^Color Trails Test 2*.

*^h^Short Category Test*.

**Table 3:** **Multivariate, unstandardized path coefficients, standard errors, 95% confidence intervals, and *p*-values (*N* = 370)**.

		Coefficient	SE	95% CI	*P*
Alcohol days^a^	AVLT^f^	−0.01	0.03	−0.07, 0.51	0.79
	CTT2^g^	−0.05	0.16	−0.36, 0.26	0.75
	SCT^h^	0.02	0.04	−0.06, 0.11	0.59
Marijuana days^b^	AVLT	0.03	0.04	−0.05, 0.11	0.45
	CTT2	0.32**	0.17	−0.001, 0.65	0.05
	SCT	−0.01	0.06	−0.13, 0.10	0.82
Cocaine days^c^	AVLT	0.00	0.03	−0.05, 0.06	0.94
	CTT2	−0.13	0.14	−0.40, 0.15	0.37
	SCT	0.02	0.05	−0.07, 0.11	0.65
Years of HIV infection^d^	AVLT	−0.04	0.10	−0.23, 0.15	0.66
	CTT2	1.18*	0.54	0.12, 2.25	0.03
	SCT	0.14	0.15	−0.17, 0.44	0.37
Time from HIV diagnosis to seeking medical care^e^	AVLT	0.18	0.38	−0.56, 0.92	0.63
	CTT2	−1.77	2.33	−6.34, 2.79	0.45
	SCT	−0.48	0.61	−1.68, 0.44	0.43

*Model included age, gender and education level as covariates*.

*^a^Number of days using alcohol in past 3 months*.

*^b^Number of days using marijuana in past 3 months*.

^c^Number of days using cocaine in past 3 months

*^d^Duration of known HIV infection in years*.

*^e^Time interval from diagnosis of HIV infection to seeking medical treatment*.

*^f^Auditory Verbal Learning Test*.

*^g^Color Trails Test 2*.

*^h^Short Category Test*.

*Significant at *p* < 0.05.

**Approaching significance.

## Discussion

Neurocognitive impairment was common (39%) in this group; however, other U.S. studies of HIV-seropositive adults using similar NC measures have yielded different results. A U.S. study of adult PLWH found their participants scored better on CTT2 ($\bar{x}=100.1$) than participants in this study (50). Another study of HIV-seropositive African-American men also reported significantly better CTT2 scores with mean score of 93.09 for their HIV^+^ symptomatic group (51). These higher functioning levels could be explained by their samples’ lower mean age and AOD use characteristics.

While not all hypothesized associations were supported in this model, two variables – marijuana use and duration of HIV infection – were associated with poorer performance on one NC measure. Albeit marginal, higher number of days of marijuana use showed some relation with poorer performance on the CTT2, an instrument that measures frontal systems functioning such as attention, executive functions, and information processing (41, 52). Although significant differences in CTT2 scores have been identified between HIV-positive intravenous drug users and non-users (53), previous research on the NC effects of marijuana use has yielded mixed results. Most studies examining marijuana-associated NC functioning found long-term use to impede performance in domains of executive functions, verbal memory, and psychomotor speed (38, 54, 55). While it is believed that chronic illicit drug use can exacerbate NC sequelae of HIV infection (25, 56, 57), little actual exploration of long-term cognitive impact of marijuana use has been conducted among PLWH. One study reported a significant association between marijuana use and poorer NC functioning among PLWH with more advanced disease, with most profound effects in the area of memory performance (58).

Methodological issues associated with NC assessment and effects of marijuana use should be considered when interpreting these and similar study outcomes (54). In this study, a 3-month drug use history was collected with no control for any recent period of drug abstinence; thus, it is possible that NC performance measures may have also captured intoxication effects of marijuana. Additionally, only associations with performance on the CTT2 measure were significant; however, this instrument may measure several domains of functioning, including psychomotor speed attention (41). Without the corroboration of instruments measuring similar NC domains, the exact areas of neuropsychological impact associated with marijuana use cannot be determined with certainty.

An additional finding of this research was that the number of years living with HIV predicted poorer performance on the CTT2 instrument; however, no significant association was found between NC functioning and the length of time from HIV diagnosis to seeking care. Although ART has significantly reduced incidence of severe NC disorders among PLWH, milder HAND remains common even among virally suppressed patients (59). In this sample, it should be noted that participants were heterogeneous, in varying stages of ART treatment and exhibiting different degrees of viral suppression. There are several possible explanations for poorer NC functioning observed among those living longer with HIV, but not necessarily associated with delayed ART initiation. An inflammatory response can occur when immune functioning rebounds after treatment with ART, known as immune reconstitution inflammatory syndrome (IRIS). However, regardless of possible IRIS, greater immunosuppression remains a strong predictor of NC impairment among PLWH (1, 60); thus, early initiation of ART is reported to be one of the most important preventive measures against developing HAND (13, 61) and continuous adherence remains a protective factor against NC impairment (17). While some participants in this study may have initiated treatment early, only about 77% of participants in this group report currently taking ART. It is also possible that the association found between years living with HIV and poorer CTT2 performance may be a function of age-related cognitive decline (62); although the analyses controlled for age.

Certain limitations of this study are important to note. First, the tested model was just identified and future studies may seek to assess models where non-significant paths are trimmed as one avenue for replicating these study findings. Also, reporting bias should be considered in interpreting outcomes. Nearly a quarter of the study population was in abstinence-based, residential addiction treatment at the time of this study, and it is possible that recent substance use was underreported. Residential treatment status was not a covariate in this model primarily because all participants had a history of substance use and frequently utilized substance abuse services – both inpatient and outpatient. While residential treatment status could have been responsible for underreporting of recent substance use, including this variable in the model could have created excessive noise. Moreover, underreporting may have varied by substance; marijuana use is likely to be less stigmatized than cocaine use (63) and possibly less than alcohol use. Measurement issues also should be considered. HIV or drug/alcohol use can affect other cognitive functions not evaluated here, such as prospective memory and cognitive impulsivity (16, 64, 65). The NC instruments used, however, are commonly utilized in similar research and considered to be reliable. Of greater concern is that poor mean NC scores for this group may also suggest poorer recall on self-report measures. In addition, the CTT2 may be non-specific, evaluating several domains of functioning. Finally, the measurement of time from HIV diagnosis to seeking care did not provide information on actual time to ART initiation. For the purpose of this study, the measure was used as a proxy for ART initiation – i.e., one who sought care sooner might also be treated with ART sooner.

Counter to reports that cocaine and alcohol use exacerbate NC impairment among PLWH (24, 26, 66, 67), this particular study did not find such associations. Because abuse of alcohol and illicit substances is inextricably linked to HIV (22), further investigation into interaction effects between long-term drug/alcohol use and HIV is recommended. Further, future research should consider the contributions of substance use to overall NC functioning for the delivery of clinical care and health behavior interventions. Early HIV care can help decrease potential NC impairment and yield better health outcomes for HIV-infected populations; however, people suffering from substance use disorders or cognitive impairment may find it difficult to follow important medical recommendations (17, 24, 29, 68–70). Outcomes from this study provide further evidence of the prevalence of NC impairment that can help to guide and enhance interventions for PLWH who use alcohol and drugs.

## Conclusion

Associations found with the CTT2 in particular points to the utility of this instrument for HAND assessment. In order to deliver effective health care to PLWH, especially those who use substances, it is important to understand the areas of functioning most affected by HAND and potentially incorporate into care remediation strategies that will help improve patient engagement. The delivery of health interventions and clinical care should consider taking a harm-reduction approach: working with the patient’s current substance use status to improve HIV treatment adherence and service utilization and delivering interventions and services that are sensitive to cognitive difficulties (71–73). Finally, it may be advised to consider including information about HAND into HIV prevention efforts, particularly to encourage testing and early treatment. It is unclear to date how knowledgeable at risk populations are about NC effects of HIV; however, growing evidence shows that NC impairment is lower among PLWH who are treated early (74).

## Conflict of Interest Statement

The authors declare that the research was conducted in the absence of any commercial or financial relationships that could be construed as a potential conflict of interest.
